# Rail surface defect data enhancement method based on improved ACGAN

**DOI:** 10.3389/fnbot.2024.1397369

**Published:** 2024-04-09

**Authors:** He Zhendong, Gao Xiangyang, Liu Zhiyuan, An Xiaoyu, Zheng Anping

**Affiliations:** ^1^School of Electrical and Information Engineering, Zhengzhou University of Light Industry, Zhengzhou, China; ^2^School of Rail Transit Engineering, Zhengzhou Technical College, Zhengzhou, China

**Keywords:** ACGAN, data enhancement, residual block, spectral norm regularization, gradient punishment

## Abstract

Rail surface defects present a significant safety concern in railway operations. However, the scarcity of data poses challenges for employing deep learning in defect detection. This study proposes an enhanced ACGAN augmentation method to address these issues. Residual blocks mitigate vanishing gradient problems, while a spectral norm regularization-constrained discriminator improves stability and image quality. Substituting the generator’s deconvolution layer with upsampling and convolution operations enhances computational efficiency. A gradient penalty mechanism based on regret values addresses gradient abnormality concerns. Experimental validation demonstrates superior image clarity and classification accuracy compared to ACGAN, with a 17.6% reduction in FID value. MNIST dataset experiments verify the model’s generalization ability. This approach offers practical value for real-world applications.

## 1 Introduction

Rail tracks serve as a crucial component of the transportation system, and the research on surface defect detection holds significant importance (Zhang et al., 2019). However, traditional detection methods are often limited by their efficiency and accuracy, making it difficult to meet the high safety standards of modern railway systems. In recent years, deep learning has made significant progress in the field of defect detection, providing a new solution for surface defect detection on railway tracks with its powerful feature extraction and pattern recognition capabilities. Nevertheless, the success of deep learning methods often depends on three key factors: computational power, datasets, and algorithms. Among these, obtaining sufficient datasets for defect detection remains a significant challenge. The scarcity and complexity of railway track defect samples can hinder the convergence of models during training, affecting their stability and compromising the accuracy of surface defect detection on railway tracks (An et al., 2023). Therefore, addressing the issue of insufficient samples is crucial for achieving effective data augmentation and enhancing the performance of deep learning models in this domain.

Image-based data augmentation methods are primarily categorized into two types: traditional machine vision techniques and machine learning approaches (Jia et al., 2023). Traditional machine vision methods typically employ geometric transformations, color conversions, and pixel manipulations (Li et al., 2023; Zhang et al., 2023). While these techniques can alleviate the issue of overfitting in neural networks to some extent, they fail to fundamentally address the challenge of insufficient sample size. Within machine learning methods, generative models (Gao et al., 2022) have garnered significant attention due to their ability to produce more diverse samples. Goodfellow et al. (2020) introduced the Generative Adversarial Network (GAN), which revolutionized image generation. However, GANs often suffer from convergence issues as they rely solely on the discriminator’s ability to distinguish between real and fake samples and are sensitive to initial parameter settings. Additionally, their inputs are limited to random noise and real samples. As GANs evolved, numerous improved models emerged. One such model is the Conditional Generative Adversarial Net (CGAN)(Tang et al., 2020) which incorporates conditional constraints (such as class labels) into both the generator and discriminator to guide the data generation process. Despite its advancements, CGAN still faces challenges such as unstable training and poor image quality (Zhang et al., 2021). The introduction of the Deep Convolution Generative Adversarial Network (DCGAN) (Rasheed et al., 2023) marked progress in reducing the blurriness of generated images. Nonetheless, it struggles with issues like mode collapse and convergence difficulties. Guo et al. (2022) proposed a structure that combines Convolutional Neural Networks (CNN) with DCGAN for accelerated self-diagnosis of sensor faults and self-recovery of fault signals. This approach enhances the accuracy of fault signals and exhibits better noise resistance. Although these methods utilize algorithms to aid image generation, they often suffer from low image quality due to their limited focus on addressing gradient vanishing problems.

To address the issue of gradient vanishing in Generative Adversarial Networks (GANs), Martin Arjovsky et al. (2017) introduced the Wasserstein GAN (WGAN), which replaces the Jensen-Shannon (JS) divergence and Kullback–Leibler (KL) divergence with the Wasserstein distance. However, this approach suffers from weight polarization, potentially leading to gradient explosion. Gulrajani et al. (2017) proposed the WGAN-GP model, which mitigates the gradient explosion issue in WGAN by using gradient penalty instead of weight clipping. Nevertheless, the model still struggles with generating high-quality images. Mao et al. (2017) presented the Least Squares Generative Adversarial Networks (LSGAN), replacing the GAN loss function with a least squares loss function to alleviate issues of unstable training and poor image quality. However, training instability remains a concern. Niu et al. (2021) introduced a novel GAN architecture based on adaptive pyramid graphs and variant residuals, aiming to enhance the detection of weak texture anomalies by generating more abnormal images and reducing the need for manual annotations. However, this network also faces gradient vanishing challenges. Wu et al. (2020) designed the ResMask GAN, which includes global and local discriminators, along with a coarse-to-fine module that seamlessly integrates generated defects into the background, achieving impressive detection accuracy even with limited samples. However, its generated results lack classification capabilities. Zhang et al. (2022) proposed the Multi-Scale Progressive Generative Adversarial Network (MAS-GAN), combining non-leaking data augmentation and a self-attention mechanism to synthesize surface defect images for assisting deep learning-based object detection algorithms. While capable of generating multi-scale defect images, the image quality remains low. Odena et al. (2017) introduced the Auxiliary Classifier Generative Adversarial Network (ACGAN), which can determine the class of generated images but is prone to mode collapse. Li et al. (2022) presented the Modified Auxiliary Classifier GAN (MACGAN), incorporating the Wasserstein distance into a new loss function to overcome mode collapse and gradient vanishing. Spectral normalization is used to replace weight clipping, constraining the discriminator’s weight parameters. This approach significantly improves the accuracy and stability of generated samples. In summary, most GAN models struggle with gradient vanishing issues, leading to mode collapse and affecting the diversity and clarity of generated images. Addressing these challenges, this paper proposes an improved ACGAN method to mitigate gradient vanishing, achieve data augmentation, and enhance image quality. The main contributions of this work are summarized as follows:

1. We improve the network structures of both the generator and discriminator by introducing residual blocks and Spectral Norm Regularization (SNR) to optimize issues related to gradient vanishing and abnormal gradient changes. Additionally, we replace the deconvolution in the generator with upsample followed by convolution and incorporate downsample layers in the discriminator to reduce computational complexity.
2. We enhance the network’s loss function by treating the discrimination task in GANs as a Positive-Unlabeled (PU) learning approach. Furthermore, we incorporate a gradient penalty mechanism based on the minimax regret method to constrain the magnitude of the discriminator’s gradient changes, enabling the network to focus more on generating image quality.

The refined model not only elevates the quality of generated images but also effectively mitigates issues like gradient vanishing and mode collapse. Initially, this paper briefly introduces the fundamental concepts of Generative Adversarial Networks and Conditional Generative Adversarial Networks. Subsequently, it delves into the optimizations made to the network architecture and objective function within the proposed enhanced model. To substantiate the efficacy of our refined model, a series of experiments are conducted on a dataset of rail surface defects in the third section. Comparative results with traditional ACGAN methods demonstrate improvements in terms of generated image quality and diversity. Finally, we conclude by summarizing the entire study.

## 2 Related work

### 2.1 Generative adversarial network

**Figure 1:**
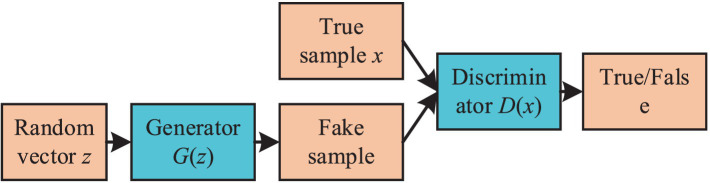
The basic structure diagram of GAN.

The concept of GAN is inspired by zero-sum games, a type of non-cooperative game theory. In the context of neural networks, this translates into a generator producing samples and a discriminator assessing their authenticity. These two components continually oppose each other, resulting in the generation of increasingly realistic samples. The ultimate goal of a GAN is to achieve a Nash equilibrium between its two networks. As illustrated in Figure 1, a GAN primarily consists of a generator and a discriminator, often implemented using Convolutional Neural Networks (CNNs) or Multilayer Perceptrons (MLPs). The primary objective of a GAN is to attain the optimal solution for its optimization function, GAN’s optimization function is shown in Equation 1:

(1) $$\begin{array}{c} \max_{D}\min_{G}V\left(D,G\right)=E_{x\sim P_{r}}\left[\log D\left(x\right)\right]\\ \;+E_{z\sim P_{z}\left(z\right)}\left[\log\left(1-D\left(G\left(z\right)\right)\right)\right] \end{array}$$

Where *x* represents an image, *z* is a sample from the latent space, *G* is the generator, *D* is the discriminator, *P_r_* denotes the distribution of real samples, and *P_z_* represents the distribution of samples in the latent spac.

### 2.2 ACGAN

**Figure 2:**
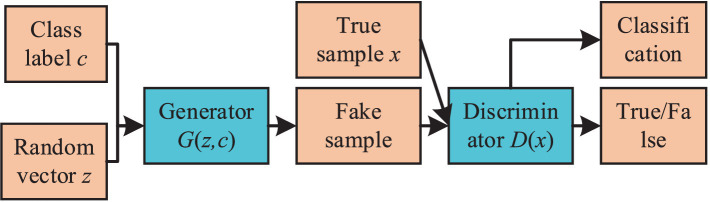
The basic structure diagram of ACGAN.

In contrast to standard GAN architectures, ACGAN integrates both a noise input z for its generator component and a categorical label constraint c in its generative process. This dual approach aims to direct the generator toward producing samples of a specified category. Meanwhile, the discriminator in ACGAN performs dual functions: it not only distinguishes the authenticity of the samples but also contributes to their classification. The fundamental structure of ACGAN is depicted in Figure 2.

The loss function of ACGAN consists of both discriminative loss and classification loss. The discriminative loss (*L_dis_*) is utilized to discern between genuine and fake samples. The classification loss (*L_cla_*) gages the accuracy of the output sample categories, as shown in Equation 2:

(2) $$L_{cla}=E_{x\sim P_{r}}\left[L_{D}\left(x|c_{x}\right)\right]+E_{z\sim P_{z}\left(z\right),c\sim P_{c}}\left[L_{D}\left(G\left(z,c\right)|c\right)\right]$$

Where *L_D_* is the category loss function, *c_x_* represents the category label, *c ~ P_c_* is the category label for the generated sample, and *P_c_* is the distribution of category labels. The loss function *L* (*D*) for the discriminator D is given by:

(3) $$L_{\mathrm{dis}}\left(D\right)=E_{x\sim P_{r}}\left[\log D\left(x\right)\right]+E_{z\sim P_{z}\left(z\right),c\sim P_{c}}\left[\log\left(1-D\left(G\left(z,c\right)\right)\right)\right]$$

(4) $$L\left(D\right)=L_{cla}+L_{\mathrm{dis}}\left(D\right)$$

The loss function *L* (*G*) for the generator *G* is:

(5) $$L_{\mathrm{dis}}\left(G\right)=E_{z\sim P_{z}\left(z\right),c\sim P_{c}}\left[\log\left(1-D\left(G\left(z,c\right)\right)\right)\right]$$

(6) $$L\left(G\right)=L_{cla}+L_{\mathrm{dis}}\left(G\right)$$

In fact, Equations 3, 5 are optimization functions for the discriminator and generator, respectively. Equations 4, 6 are generation functions for the discriminator and generator, respectively. In the end, ACGAN is designed to both generate high-quality samples and classify them.

## 3 Improved ACGAN

**Figure 3:**
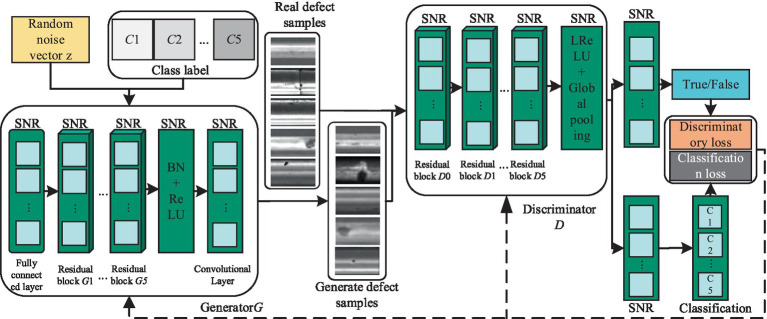
The basic structure diagram of the improved ACGAN.

ACGAN represents an advancement over GAN by incorporating image classification abilities while simultaneously enhancing the quality of generated samples. Nevertheless, when utilized for generating samples of rail surface defects with limited datasets, ACGAN may encounter obstacles such as subpar sample quality, unstable model training, and mode collapse. To mitigate these challenges, this paper introduces an enhanced ACGAN model, depicted in Figure 3. In this refined model, a random noise vector, along with the category label, serves as input to the generator. Both the real samples and those generated by the generator are then fed into the discriminator, tasked with not only distinguishing authenticity but also performing classification. Subsequently, the optimization of this ACGAN model is approached comprehensively, addressing both network architecture and objective functions.

### 3.1 Optimization of the network model

**Figure 4:**
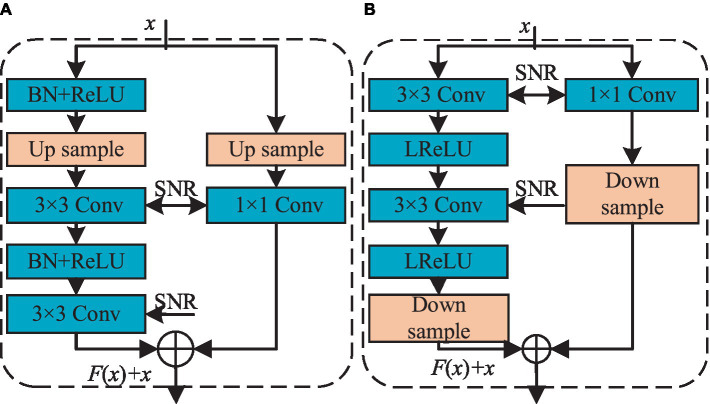
The basic structure diagram of residual block. **(A)** Residual blockG; **(B)** Residual blockD.

To address lingering issues like gradient vanishing in the ACGAN network, this paper introduces improvements from the perspective of optimizing the network structure. The specific methods are as follows:

1. To alleviate the gradient vanishing issue, residual blocks have been incorporated into both the generator and discriminator. These blocks elevate network performance, enhancing the generator’s sample generation capabilities, bolstering the discriminator’s ability to distinguish between real and fake samples, and facilitating classification. Figure 4 depicts the residual structure employed in both the generator and discriminator. The Tanh function is employed in the generator’s output layer, while the remaining layers utilize the “Batch Normalization (BN) + ReLU” configuration, coupled with residual blocks to train deeper networks effectively. In the discriminator, LeakyReLU activation functions are used throughout all layers to mitigate the “neuron death” issue commonly associated with the ReLU function.
2. Incorporating SNR (Zhang et al., 2019) into both the generator and discriminator enhances model performance. During extensive network training, models may converge toward Sharp Minimizers, compromising their generalization capabilities. Spectral norm regularization ensures that the weight matrices utilized by neural networks maintain a controlled spectral norm, thus mitigating this issue. By leveraging the SNR method, the trained model’s sensitivity to perturbations in test data is diminished. High sensitivity at local minima negatively impacts the generalization performance of the model. Therefore, based on the premise that flatter local minima equate to stronger generalization abilities, this paper establishes a correlation between local flatness and singular values, leading to the introduction of SNR. Specifically, in neural networks, regularization constraints are imposed from the perspective of the spectral norm of each layer, preventing abnormalities such as rapid parameter growth and gradient fluctuations in the generator. Input perturbation for neural networks *ξ*, The calculation for measuring disturbances is shown in Equation 7:

(7) $$\begin{array}{l} \frac{∥f_{\Theta }\left(x+\xi \right)-f\left(x\right){∥}_{2}}{∥\xi {∥}_{2}}\\ =\frac{∥\left(W_{\Theta ,x}\left(x+\xi \right)+b_{\Theta ,x}\right)-\left(W_{\Theta ,x}x+b_{\Theta ,x}\right){∥}_{2}}{∥\xi {∥}_{2}}\\ =\frac{∥W_{\Theta ,x}\xi {∥}_{2}}{∥\xi {∥}_{2}}\leq \sigma \left(W_{\Theta ,x}\right) \end{array}$$

Among them, *f_Θ_* Represents the nonlinear activation function, *x* represents the input, using *W_Θ_,_x_x + b_Θ_, x* as an affine mapping to represent *f_Θ_*, the spectral norm of *x*, for a matrix A, it is defined as shown in Equation 8:

(8) $$\sigma \left(A\right)=\max_{\xi \in R^{n},\xi \neq 0}\frac{∥A\xi {∥}_{2}}{∥\xi {∥}_{2}}$$

The spectral norm is the maximum singular value of matrix A, therefore, if *W_Θ_*, If the spectral norm of *x* is maintained at a small value, then *f_Θ_* Will be insensitive to disturbances in *x*. To limit *W_Θ_*, the spectral norm of *x* is achieved by adding a regularization term to the loss function:

(9) $$\underset{\Theta }{\mathrm{minimize}}\frac{1}{K}\overset{K}{\underset{i=1}{\sum }}L\left(f_{\Theta }\left(x_{i}\right),y_{i}\right)+\frac{\lambda }{2}\overset{L}{\underset{𝓁=1}{\sum }}\sigma {\left(W^{𝓁}\right)}^{2}$$

Where l represents the l-th layer of the neural network.

**Figure 5:**
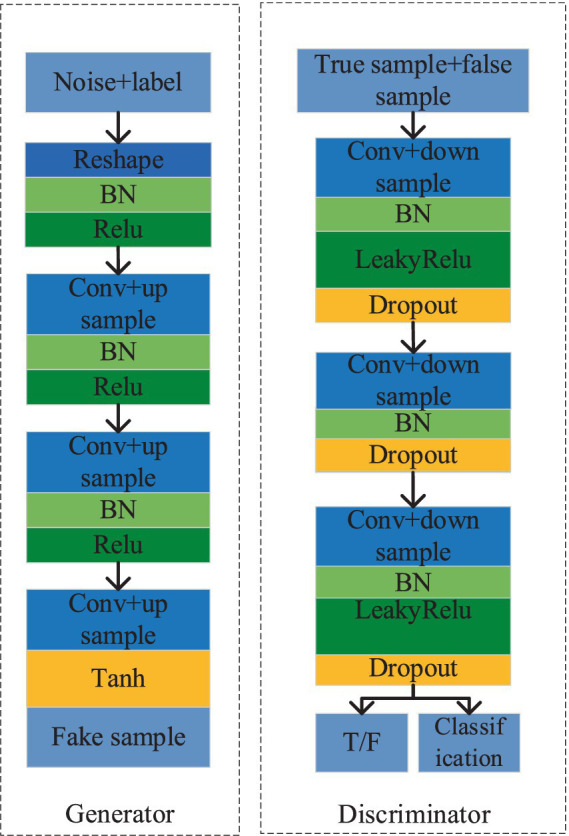
The modified generator and discriminator network structure diagram.

1. This article replaces the deconvolution layer in the generator with a combination of upsample and convolution, while constructing the discriminator using downsample convolution. The modified structures of both the generator and discriminator are depicted in Figure 5, with the generator on the left side and the discriminator on the right. Specifically, the generator employs nearest neighbor interpolation for upsample, effectively doubling the feature map and mitigating the checkerboard artifact commonly associated with deconvolution operations. Conversely, the discriminator incorporates an average pooling operation with a stride of 2, reducing computational complexity and expanding the receptive field range.

### 3.2 Optimization of the objective function

Traditional ACGAN disregards variations in the quality of generated samples, leading to unstable model training, imbalanced sample generation, and ultimately affecting classification accuracy. To mitigate these issues, this paper approaches the problem by classifying positive and unlabeled samples, treating the discrimination of real and fake samples in ACGAN as a Positive-Unlabeled (PU) learning technique. Under this framework, the distribution of generated samples can be expressed as:

(10) $$P_{q}\left(x\right)=\delta P_{hq}\left(x\right)+\left(1-\delta \right)P_{lq}\left(x\right)$$

Where *P_hq_* (*x*) and *P_lq_* (*x*) represent the probability distributions of high-quality and low-quality generated samples, respectively, and *δ* indicates the proportion of high-quality samples in the total generated samples.

In ACGAN, the discriminator is tasked not only with discerning the authenticity of samples but also distinguishing between high and low-quality generated samples. Assuming *δ* is given, the objective function for the discriminative loss will become:

(11) $$\begin{array}{c} L_{\mathrm{dis}}\left(D\right)=\delta E_{x\sim P_{hq}}\left[\log D\left(x\right)\right]\\ \;+\left(1-\delta \right)E_{x\sim P_{lq}}\left[\log\left(1-D\left(x\right)\right)\right] \end{array}$$

The expectation *E_x ~ Phq_* in the formula represents the expected value, where *x* is sampled from the high-quality distribution *P_hq_*.

Considering high-quality samples and real samples as one category, where a high-quality sample *x_hq_* can be represented by a real sample *x_r_*, the distribution of low-quality generated samples, based on Equation, 10, is represented as:

(12) $$\left(1-\delta \right)P_{lq}\left(x\right)=P_{q}\left(x\right)-\delta P_{r}\left(x\right)$$

In Equation, 12, the target data distribution *P_q_ (x)* can be represented as a weighted combination of the low-quality data distribution *P_lq_ (x)* and the reference data distribution *P_r_ (x)*.

According to Equations 11, 12, the objective function for the discriminator is:

(13) $$\begin{array}{c} L_{\mathrm{dis}}\left(D\right)=\delta E_{x\sim P_{r}}\left[\log D\left(x\right)\right]\\ \;+\max\left\{\begin{array}{c} 0,E_{z\sim P_{z},c\sim P_{c}}\left[\log\left(1-D\left(G\left(z,c\right)\right)\right)\right]\\ -\delta E_{x\sim P_{r}}\left[\log\left(1-D\left(x\right)\right)\right] \end{array}\right\} \end{array}$$

Where the max{} function is used to prevent the occurrence of negative loss functions.

Equation 9 categorizes the discriminator’s output into low and high-quality samples, assigning higher weights to the latter to encourage the generator’s production of superior samples. Nonetheless, this approach may result in the GAN synthesizing numerous similar high-quality samples, leading to excessive homogeneity. Simultaneously, the discriminator’s gradient is susceptible to exploding, destabilizing the network and hindering effective data augmentation. To mitigate these challenges, this paper incorporates Spectral Norm Regularization, which stabilizes the Generative Adversarial Network (GAN) by imposing constraints on the spectral norm of each discriminator layer. This method boasts a lower computational cost and obviates the need for hyperparameter tuning compared to alternative techniques. Furthermore, a gradient penalty mechanism rooted in the max-min regret method is integrated into the discriminator’s objective function. Originating from two-player zero-sum games (Liu et al., 2021), the max-min regret method computes the Nash equilibrium, thereby limiting the magnitude of the discriminator’s gradient variations. The corresponding formula is presented as follows:

(14) $$L_{GP}=E_{x\sim P_{r},\varphi \sim U_{r}}\left(0,\sigma_{x}\right){\left[\nabla_{x}D\left(x+\varphi \right)-1\right]}^{2}$$

Where *φ* represents the noise information.

The improved generator discriminative loss function for ACGAN is:

(15) $$L_{\mathrm{dis}}\left(G\right)=E_{z\sim P_{z}\left(z\right)}\left[\log\left(1-D\left(G\left(z\right)\right)\right)\right]$$

In addition to the discriminative loss, the category loss also needs further optimization. The classification loss for real and generated samples is separated, and the discriminator and generator are optimized separately. Ultimately, by using Equations 13, 14, and the calculation of the discriminator’s objective function *L* (*D*) is calculated as shown in Equation 16:

(16) $$L\left(D\right)=L_{\mathrm{dis}}\left(D\right)+\lambda L_{GP}+\gamma E_{x\sim P_{r}}\left[L_{D}\left(x|c_{x}\right)\right]$$

Where *λ* is the weight of the gradient penalty, and *γ* is the proportion of the total loss function represented by the classification loss. The generator’s objective function *L* (*G*), composed of Equation 15 and the category loss for generated samples, The specific calculation is as shown in Equation 17:

(17) $$L\left(G\right)=L_{\mathrm{dis}}\left(G\right)+\gamma E_{z\sim P_{z},c\sim P_{c}}\left[L_{D}\left(G\left(z,c\right)|c\right)\right]$$

## 4 Experiments and analysis

### 4.1 Experimental dataset and experimental environment settings

**Figure 6:**
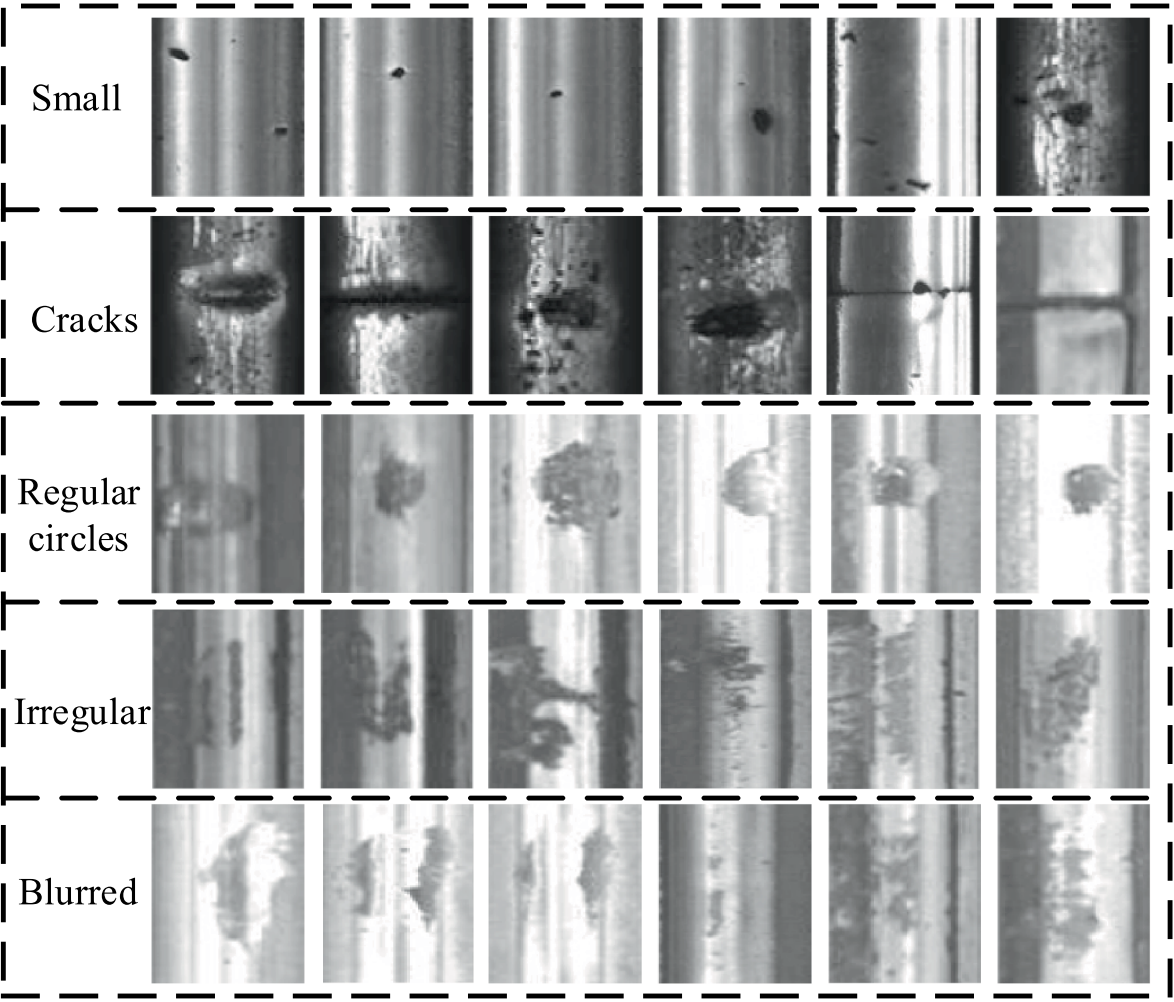
The types of rail defects.

This article utilizes a dataset specifically tailored for rail defects in its experimental endeavors. This comprehensive dataset encompasses images showcasing various rail surface imperfections (Yu et al., 2018), ensuring the inclusion of at least one defect in each image. The dataset’s visual representations originate from both fast tracks and regular/heavy tracks, offering a diverse range of scenarios. The defective images can be neatly categorized into five distinct groups: cracks, regular circles, irregular shapes, small points, and blurred areas, as depicted in Figure 6. Specifically, crack defects are characterized by narrow fissures traversing the steel rail’s surface; circular defects denote imperfections in the form of circles on the track’s surface; irregular defects encompass those potentially arising from numerous fine-grained shapes; small point defects pertain to minute surface blemishes on steel rails, discernible upon image enlargement; and fuzzy defects refer to those where the rail defect’s outline is indistinct to the naked eye.

This study conducted the training of network models in a hardware environment equipped with an AMD Ryzen 75800H with Radeon Graphics processor, an NVIDIA GeForce RTX 3050 graphics card, 16GB of memory, and a Windows operating system. To ensure the accuracy and objectivity of the research, we employed the PyTorch deep learning framework and trained all network models using identical parameter settings. During the training process, all models underwent 200 epochs of iteration on the same dataset, maintaining a batch size of 8 to guarantee a fair comparison and prevent biases in the training process.

### 4.2 Effect of dataset-generated samples

**Figure 7:**
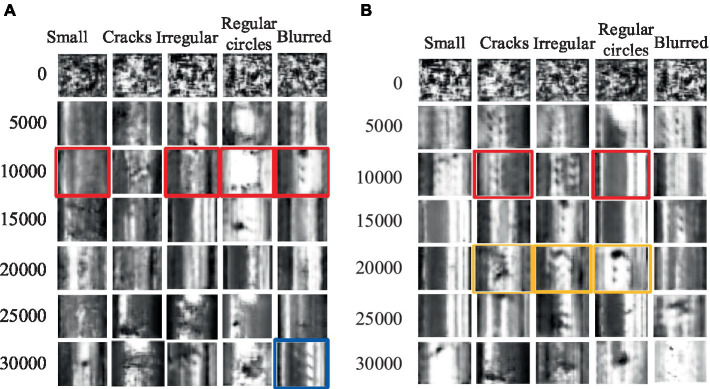
The sample generation effect of rail surface defects. **(A)** ACGAN; **(B)** Improved ACGAN.

Firstly, experiments were conducted using the rail surface defect dataset. Figure 7 presents an example of generated rail surface defect samples, with Figure 7A showcasing the performance of ACGAN and Figure 7B exhibiting the results of our model after 5,000 rounds of training. Ideally, the image categories should progress from small defects in the first column to cracks, irregular circles, regular circles, and blurry defects in the subsequent columns, respectively. From the graphs, it is evident that both our proposed model and the ACGAN exhibit image blurring and misclassification during the initial stages when the training rounds are less than 10,000. In Figure 7A, the ACGAN’s training results after 10,000 rounds reveal mismatches in the preset types for the small, irregular circle, regular circle, and fuzzy classes, marked in red. Similarly, in Figure 7B, our model’s results after the same number of rounds indicate mismatches in the preset types for the crack and regular circle classes, also marked in red. As the number of training iterations increases to 20,000, the ACGAN’s training results in Figure 7A still do not allow for a specific distinction of defect types. However, in Figure 7B, despite some blurriness, our model can already distinguish between crack, irregular circle, and regular circle defects, marked in orange boxes. When the training rounds reach 30,000, both our model and ACGAN generate defect images that are somewhat blurry compared to the original dataset. Nevertheless, our model can clearly distinguish all five types of defect images. In contrast, ACGAN’s generated fuzzy defect images exhibit classification errors, marked with blue boxes in Figure 7A. By comparing the display effects, it is evident that our proposed model demonstrates superior generation performance compared to ACGAN.

**Figure 8:**
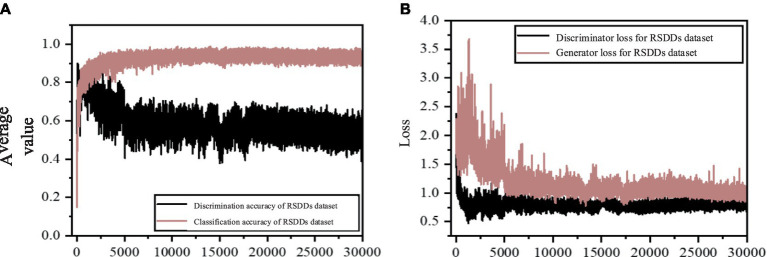
The mean accuracy and loss value maps of improved ACGAN in rail defect dataset. **(A)** Average precision graph for the improved ACGAN; **(B)** Loss value graph for the improved ACGAN.

Figure 8 illustrates the average accuracy and loss values of the model presented in this study, utilizing the steel rail surface dataset. In Figure 8A, the classification accuracy curve demonstrates a sharp ascent prior to 2000 rounds, succeeded by a gradual increase between 2,500 and 5,000 rounds. Subsequently, after 5,000 rounds, the curve stabilizes within the range of 0.85 to 0.95. Notably, the Nash equilibrium, a dynamic state of balance, is evident in the curve’s pattern. Despite experiencing fluctuations, it consistently returns to a value around 0.9, suggesting that this approximate value represents a temporary equilibrium solution. Similarly, the discriminator’s accuracy curve initially rises slightly, then declines, and stabilizes between 0.5 and 0.7 by approximately 20,000 rounds. It attains a temporary equilibrium at a value of approximately 0.6, further indicating the model’s attainment of Nash equilibrium. Turning to Figure 8B, the generator loss curve exhibits a slight increase followed by stabilization, whereas the discriminator loss curve progressively decreases and stabilizes. These patterns collectively suggest that the model exhibits strong convergence performance.

Upon comparing the results obtained from the steel rail defect dataset, it is evident that the enhanced model introduced in this study surpasses ACGAN in terms of sample generation clarity and classification accuracy. Examination of the average accuracy chart and loss value chart for the steel rail defect dataset reveals that the refined model steadily enhances and stabilizes the classification accuracy curve as training rounds increase, without any notable decline in accuracy. Additionally, the absence of significant fluctuations in the loss curve during the loss update indicates that the model effectively addresses the issues of gradient disappearance and gradient anomaly.

### 4.3 Comparison of FID values

To comprehensively evaluate the quality of samples generated by the enhanced ACGAN model, this study employs the Fréchet Inception Distance (FID)(Heusel et al., 2017) as the core evaluation metric, which is widely recognized in GAN performance assessments. FID quantifies the dissimilarity between the feature vectors of real and generated samples, with a lower FID value indicating superior image quality. The choice of FID as the core indicator for image quality assessment is not only based on its solid theoretical foundation but also on its significant advantages in practical applications. It effectively captures the perceptual similarity between images, providing a comprehensive and in-depth evaluation of the model’s generative capabilities. By leveraging FID, we aim to reveal more accurately the performance of the enhanced ACGAN model in generating high-quality samples.

When comparing ACGAN with our proposed model using the steel rail defect dataset, the FID values presented in Table 1 show a consistent decrease across all categories for our model. Specifically, fuzzy defects, which exhibit complex features, are better captured by our model, leading to a more pronounced reduction in FID values compared to ACGAN. Conversely, small defects with more obvious features are easily generated by both models, resulting in a lesser decrease in FID values. Overall, our model achieves an average FID reduction of 17.6% compared to ACGAN, indicating a significant improvement in the quality of generated samples.

**Table 1:** FID values for each type of rail surface defect dataset.

Class	Blurred	Cracks	Irregular	Regular circles	Small	Average value
ACGAN	352.1	178.6	248.6	206.2	153.2	227.7
Improved ACGAN	254.8	156.8	212.5	168.4	146.5	187.5

### 4.4 Comparative experiment

**Figure 9:**
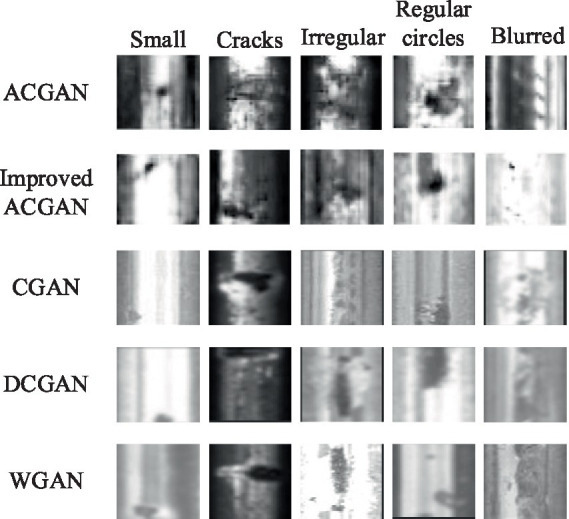
Comparison of sample generation effects for surface defects on steel rails.

To assess the effectiveness of the model presented in this study, we conducted experiments using commonly employed generative adversarial networks such as CGAN, DCGAN, and WGAN, alongside ACGAN. A comparative analysis of the generation outcomes for surface defect samples on steel rails is depicted in Figure 9. As evident from the figure, CGAN struggles with the irregular circle class, DCGAN exhibits a poor generation effect for the fuzzy class, and WGAN falters in the regular circle class, all deviating from established defect patterns. Furthermore, due to gradient anomalies arising during the training process of these three networks as training rounds increase, early improvements in image clarity do not correlate with the number of training rounds. Consequently, the resulting images appear blurry, hindering accurate identification of the generated defect types.

The comparison of FID values between our model and CGAN, DCGAN, and WGA is shown in Table 2. From the table, it can be seen that our model has the lowest FID values in various categories compared to other models. The performance of fuzzy defects is the best among them. From the average data, it can be seen that the model in this article is lower than the other three models, indicating that the sample quality generated by the model in this article is better.

**Table 2:** FID values for each model.

Class	Blurred	Cracks	Irregular	Regular circles	Small	Average value
Improved ACGAN	254.8	156.8	212.5	168.4	146.5	187.5
CGAN	362.3	192.4	271.3	222.7	176.2	245.2
DCGAN	321.5	179.6	243.8	213.5	166.9	225.1
WGAN	297.6	171.2	236.8	196.3	158.4	212.1
ACGAN	352.1	178.6	248.6	206.2	153.2	227.7

### 4.5 Generalization experiment

To assess the generalized performance of the enhanced model in this study, we conducted experiments using the MNIST handwritten dataset (LeCun et al., 1998). The selection of the MNIST dataset stemmed from its widespread utilization and established reputation in machine learning, especially for benchmarking image processing and recognition models. This dataset encompasses labels for 10 digits ranging from 0 to 9, presenting a diverse and challenging collection of handwritten samples. By leveraging the MNIST dataset, we aimed to provide a comprehensive and reliable evaluation of the model’s generalization abilities.

**Figure 10:**
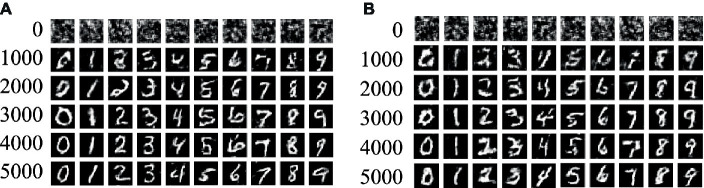
The sample generation effect of MNIST dataset. **(A)** Improved ACGAN; **(B)** ACGAN.

Figure 10 illustrates the performance of the generated MNIST handwritten digit samples. Specifically, Figures 10A,B depict the outcomes of our model and ACGAN, respectively, after 1,000 training rounds. It is evident from the figure that our model can produce clear images even at 5,000 rounds, without exhibiting issues such as blurring or font adhesion. Conversely, ACGAN exhibits noticeable image blurring and font adhesion issues even after 5,000 rounds, particularly pronounced in the digits 2, 3, 6, and 8. These experimental findings demonstrate a substantial improvement achieved by our proposed model compared to ACGAN.

**Figure 11:**
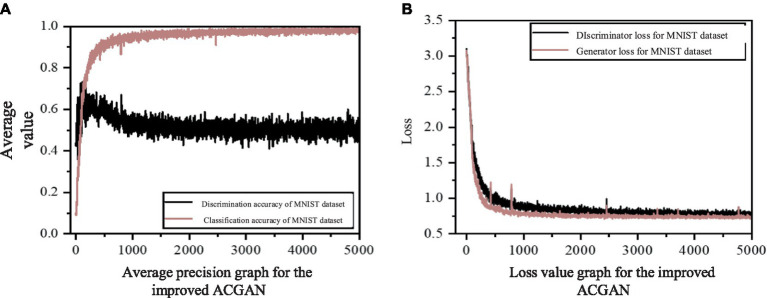
The mean accuracy and loss value maps of improved ACGAN in MNIST dataset.

Figure 11 presents the mean accuracy and loss values of the model proposed in this study, evaluated on the MNIST handwritten dataset. As observed in Figure 11A, the discrimination accuracy approaches unity at around 1,500 rounds and remains consistent thereafter. Initially, the classification accuracy experiences a slight increase, followed by a decrease to approximately 0.5 after 1,500 rounds, settling at a point near 0.5, indicating that the model has attained a Nash equilibrium state. Furthermore, in Figure 11B, the loss values for both the generator and discriminator exhibit a rapid decline and stabilize after approximately 1,500 rounds, signifying excellent convergence performance of the network model.

Upon comparing Figure 8A with Figure 11A, it becomes evident that the enhanced model exhibits superior performance on the MNIST dataset as compared to the rail defect dataset. This disparity arises from the fact that the MNIST dataset boasts clearer images and greater contrast between foreground and background elements than the steel rail defect dataset. Furthermore, the MNIST dataset’s image complexity is notably lower than that of the steel rail defect dataset. Moreover, a comparison of the average FID values presented in Tables 1, 3 reveals that the FID values associated with the MNIST dataset are considerably smaller than those of the rail surface defect dataset. This difference stems from the simpler features and higher contrast inherent in the MNIST dataset. A closer examination of the FID values across both datasets indicates that our model achieves a lower FID value than ACGAN, suggesting that our model produces images of superior quality. Additionally, these findings underscore the versatility of the model proposed in this article, demonstrating its suitability for generating tasks across multiple datasets and highlighting its impressive generalization capabilities.

**Table 3:** FID values of MNIST dataset.

Class	0	1	2	3	4	5	6	7	8	9	Average value
ACGAN	211	238	195	187	199	208	196	165	156	165	192
Improved ACGAN	16	42	60	63	60	78	49	31	21	25	46

Table 3 further illustrates the comparison of FID values between ACGAN and our model specifically on the MNIST dataset. It is apparent from Table 3 that our model consistently exhibits reduced FID values across various categories. On average, our model achieves a notable 76% reduction in FID values compared to ACGAN, indicating a significant improvement in generating samples with simpler features.

## 5 Conclusion

This article introduces an enhanced ACGAN approach tailored for augmenting rail surface defect data. This method can generate defect images that correspond to specific input categories. Building upon the foundation of ACGAN, we have incorporated residual blocks and spectral norm regularization into both the generator and discriminator networks. These additions effectively address gradient vanishing and anomaly issues, thereby bolstering the network’s stability. Furthermore, we have replaced the deconvolution method in the generator with a combination of upsample and convolution, while the discriminator employs downsample to reduce the overall computational burden. We view the authenticity discrimination problem in GANs through the lens of PU learning, assigning weights to high-quality samples to prioritize image quality during generation. Additionally, a gradient penalty mechanism, rooted in the maximum and minimum regret value method, has been integrated into the discriminator loss function to constrain gradient changes. To evaluate the effectiveness of our approach, we conducted experiments using a dataset of steel rail surface defects, assessing both the generation quality and FID values. The results are compelling: compared to ACGAN, our model produces images of superior quality without any classification errors. A comparative analysis of FID values further underscores the model’s ability to generate samples that are more aligned with real-world examples, highlighting the superiority of our generated samples. Even when pitted against other popular generative adversarial networks, our model emerges as a frontrunner in terms of image quality. Beyond this, we have also validated our model’s versatility through tests on the MNIST handwritten digit dataset. The performance of this study largely depends on the quality and diversity of the training data. Additionally, it faces challenges such as high computational overhead and improper allocation of authenticity weights. Future work may focus on addressing these limitations and further enhancing the performance and applicability of this method.

## Data availability statement

Publicly available datasets were analyzed in this study. This data can be found at: http://icn.bitu.edu.cn/Visint/resources/RSDDs.aspx and http://m6z.cn/61EkKL.

## Author contributions

HZ: Conceptualization, Funding acquisition, Investigation, Methodology, Project administration, Resources, Supervision, Validation, Visualization, Writing – original draft, Writing – review & editing. GX: Conceptualization, Investigation, Methodology, Resources, Software, Visualization, Writing – original draft, Writing – review & editing. LZ: Data curation, Resources, Writing – review & editing. AX: Formal analysis, Funding acquisition, Resources, Writing – review & editing. ZA: Formal analysis, Project administration, Writing – review & editing.
